# Utility of Scoring System for Screening and Early Warning of Cervical Cancer Based on Big Data Analysis

**DOI:** 10.3389/fpubh.2022.920956

**Published:** 2022-06-20

**Authors:** Dan Hou, Binjie Yang, Yangdan Li, Ming Sun

**Affiliations:** ^1^Department of Health Medicine, General Hospital of Northern Theater Command, Shenyang, China; ^2^China Medical University, Shenyang, China

**Keywords:** big data analysis, scoring system for early warning, cervical cancer screening, utility, intervention measures

## Abstract

**Objective:**

To explore the utility of the scoring system for screening and early warning of cervical cancer based on big data analysis.

**Methods:**

A total of 420 women undergoing physical examination in Shenyang from January 2021 to January 2022 were screened by convenient sampling as the study subjects. All females accepted the human papilloma virus (HPV) tests and thin-prep cytology test (TCT), a Rating Questionnaire for Screening and Early Warning of Cervical Cancer was developed, and a warning threshold was derived according to the scores of the questionnaire and the goodness of fit for the results of HPV+TCT tests. The patients were graded according to the threshold, and corresponding intervention strategies for patients of different grades were developed.

**Results:**

Among the 420 people undergoing physical examination, 92 (21.90%) obtained scores ≥8 points, and 328 (78.10%) obtained scores < 8 points; in diagnosing cervical cancer, the Rating Questionnaire for Screening and Early Warning of Cervical Cancer had an AUC value of 0.848, specificity of 97.22%, and sensitivity of 86.46%; after scientific intervention, HPV test results showed a significant decrease in both high-risk positive cases and low-risk positive cases (*p* < 0.05), and TCT results showed that there was a significant difference in the number of patients with CIN I before and after intervention (*p* < 0.05).

**Conclusion:**

The scoring system for screening and early warning of cervical cancer based on big data analysis presents certain clinical value in the clinical screening of cervical cancer, which can further improve the screening coverage, is of great significance for the diagnosis and treatment of disease, and helps physician implement hierarchical diagnosis and treatment quickly and precisely.

## Introduction

Cervical cancer is a malignant tumor disease that endangers women's health (1). Investigation and research (2) have shown that about 85% of cervical cancer patients are from developing countries, and there are approximately tens of thousands of new cervical cancer cases each year worldwide, seriously threatening women's health and life safety. According to years of basic experimental and epidemiological studies (3, 4), human papilloma virus (HPV) infection is an important factor causing the disease. Cervical cancer 3-step screening technique (step 1: cervical cytology and HPV test; step 2: colposcopy; step 3: histopathological examination) is currently the most widely used clinical diagnosis technique, and its clinical application provides an effective pathological evidence for the early diagnosis and treatment of cervical cancer in developed countries or regions (5). Early detection and treatment can effectively prevent the deterioration of the condition and minimize mortality. However, in countries and regions with relatively lagged economic and medical conditions, most cervical cancer patients are already in the advanced stage of the disease when diagnosed, leading to a lower cure rate and higher mortality. Therefore, searching for convenient and efficient means of cervical cancer screening can provide important auxiliary judgment indicators for clarifying and diagnosing the disease, and lay a strong scientific foundation for selecting clinical treatment options (6–8). There are deficiencies in every screening method in the existing medical context, and no single examination modality achieves the combination of high sensitivity with high specificity. Based on this, by reviewing a great amount of relevant materials of cervical cancer, a Rating Questionnaire for Screening and Early Warning of Cervical Cancer was developed in this trial to get the score of each patient and then develop corresponding intervention measures, thus improving the disease screening rate.

## Materials and Methods

### General Data

A total of 420 women undergoing physical examination in Shenyang from January 2021 to January 2022 were screened by convenient sampling as the study subjects. The study met the World Medical Association Declaration of Helsinki (2013) (9) and reviewed and approved by the Hospital Ethics Committee.

### Inclusion and Exclusion Criteria

Inclusion criteria: (1) Women with history of sexual life; (2) those with good understanding and could objectively and accurately fill in the questionnaire; and (3) those who voluntarily joined the study. Exclusion criteria: (1) Those who had cervical cancer and operation history; (2) those who were unable to fill in the questionnaire due to other reasons; and (3) those who were unwilling to join the study.

### Methods

#### Content of Questionnaire

By reviewing a large number of published works on the epidemiology of cervical cancer and related influencing factors, the first version of Rating Questionnaire for Screening and Early Warning of Cervical Cancer was developed, which included the following sections. (1) The general data of the study subjects, such as their age, place of residence, occupation, educational degree, marital status, and the paying method of medical treatment;

(2) Sexual habits, such as age at first sexual activity, number of sexual partners, and whether condoms were used at the time of intercourse;

(3) Personal situation, such as number of times of miscarriages, number of times of pregnancies, presence or absence of an intrauterine contraceptive device (IUD), and presence or absence of immunodeficiency diseases; and

(4) Relevant symptoms or vital signs, such as presence or absence of cervical contact bleeding, cervical erosion, or cervical polyps.

In addition to the personal information of the study subjects, a total of 20 items were scored, and the maximum score was 20 points. The scoring method was as follows: 1 point was assigned to each question (1 for each forward-rating item, and −1 for each backward-rating item), and higher scores indicated higher risk of illness of the study subjects.

#### Formation of Questionnaire

A Delphi Consultation Questionnaire was developed according to the corresponding general format. After 2 rounds of expert consultation and pre-survey to 30 females, relevant data were organized and the indicators were perfected in combination with the experts' suggestions, which then concluded that the Cronbach's α coefficient and split-half coefficient of the questionnaire were, respectively, 0.796 and 0.691, the validity index of each item level was ≥0.945, and the content validity index of the scale level was 0.950.

#### Test and TCTHPV Test

The HPV special sampler was inserted to the orificium externum isthmus of the subjects, rotated for 5 turns, and then slowly taken out and put into the HPV specimen bottle containing preserving liquid. The HPV subtyping genes chip was used to detect 26 types of HPV genes (such as HPV 16, 18, 31, 33, 35, 39, 45, 51, 52, 56, 58, 59, 6, 11, 40, 42, 43, 44, 53, 54, 55, 57, 66, 67, 73, among which 16, 18, 31, 33, 35, 39, 45, 51, 52, 56, 58, 59, 66, 68 were high-risk types, and 6, 11, 30, 42, 43, 44 were low-risk types), and all operations were conducted according to the specifications of the apparatus and reagent.

##### TCT

The sampler tip was inserted into the cervical canal and slowly rotated along the axis for 5 turns, the sampling brush tip was removed and put into the cell preservation solution, the specimen underwent standardized processing and was made into a thin-layer cell smear and fixed with 95% alcohol, and then Papanicolaou stain was performed. For diagnostic gradation of cytology, the Bethesda system (TBS) was adopted, to be specific, TCT negative, referring to no CLN was seen (negatives of intraepithelial lesions or malignancy, NILM); TCT abnormalities, such as a typical squamous cells of undetermined significance (ASC-US); low-grade squamous intraepithelial lesion (LSIL), which was graded as weak CIN (CIN I); high-grade squamous intraepithelial lesion (HSIL), which was graded as moderate and expressed CIN (CIN II and CIN III) and carcinoma *in situ* (CIS); and squamous cell carcinoma (SCC).

#### Quality Control

Each section of the investigation was under strict quality control, in the stage of designing, the scientificity and feasibility of the scheme and questionnaire applied were verified; before investigation, the researchers were trained with uniform guidelines, and then they provided guidance and correction on any queries and errors made by the study subjects when filling in the questionnaire; after the completion of the questionnaire, 2 personnel were assigned to collect and organize the questionnaires, Epidata3.1 was used for data entry, SPSS20.0 was used for statistical analysis, and an early warning threshold of 8 points for the questionnaire was derived by comparing the analytic findings and gynecological examination results.

### Early Warning Threshold

According to patients' HPV test and TCT results and the scores of the Rating Questionnaire for Screening and Early Warning of Cervical Cancer, the threshold was specified as 8 points, and detailed intervention measures were developed according to the threshold.

### Intervention Strategies

Detailed hierarchical intervention measures were developed according to the threshold. (1) Below threshold: For those who were free from infection and without prior HPV testing, testing was recommended for 3 consecutive years, and if the results were negative, testing could then be done every 2–3 years; (2) Above threshold: this group was high-risk HPV infected individuals, who should visit gynecological clinics according to the TCT result, and the effectiveness of intervention measures should be analyzed by comparing the results of TCT and HPV test before and after the intervention.

### Statistical Methods

The experimental data were statistically analyzed and processed by the software SPSS26.0, the diagnostic efficacy of the Rating Questionnaire for Screening and Early Warning of Cervical Cancer on cervical cancer was analyzed by the area under the Receiver Operating Characteristic (ROC) curve.

## Results

### Clinical Data of Study Subjects

See Table 1.

**Table 1:** Clinical data of study subjects.

**Clinical item**	**Proportion [%(*n*)]**
**Age**	
18–26 years	13.10 (55/420)
27–40 years	28.33 (119/420)
41–60 years	42.62 (179/420)
>60 years	15.95 (67/420)
**BMI value**	
<18.5	10.95 (46/420)
18.5–24.9	40.24 (169/420)
25.0–29.9	34.29 (144/420)
≥30.0	14.52 (61/420)
**Occupation**	
Employee of organizations, enterprises, public institutions	10.00 (42/420)
Service personnel	32.38 (136/420)
Farmer	20.24 (85/420)
Worker	15.00 (63/420)
Self-employed entrepreneur	16.43 (69/420)
Others	5.95 (25/420)
**Place of residence**	
Rural area	57.86% (243/420)
Urban area	42.14% (177/420)
**Educational degree**	
Junior college and above	18.81% (79/420)
Senior high school	35.00% (147/420)
Junior high school and below	39.52% (166/420)
Illiteracy	6.67% (28/420)
**Paying method of medical treatment**	
Medical insurance	54.52 (229/420)
Self-paying	45.48 (191/420)
**Family monthly income (yuan)**	
≥3,000	49.52 (208/420)
<3,000	50.48 (212/420)

### Scores of the Rating Questionnaire for Screening and Early Warning of Cervical Cancer of the Females Undergoing Physical Examination

Among the 420 female undergoing physical examination, 92 (21.90%) obtained scores ≥ 8 points, and 328 (78.10%) obtained scores < 8 points.

### Clinical Diagnostic Efficacy of the Rating Questionnaire for Screening and Early Warning of Cervical Cancer

**Figure 1:**
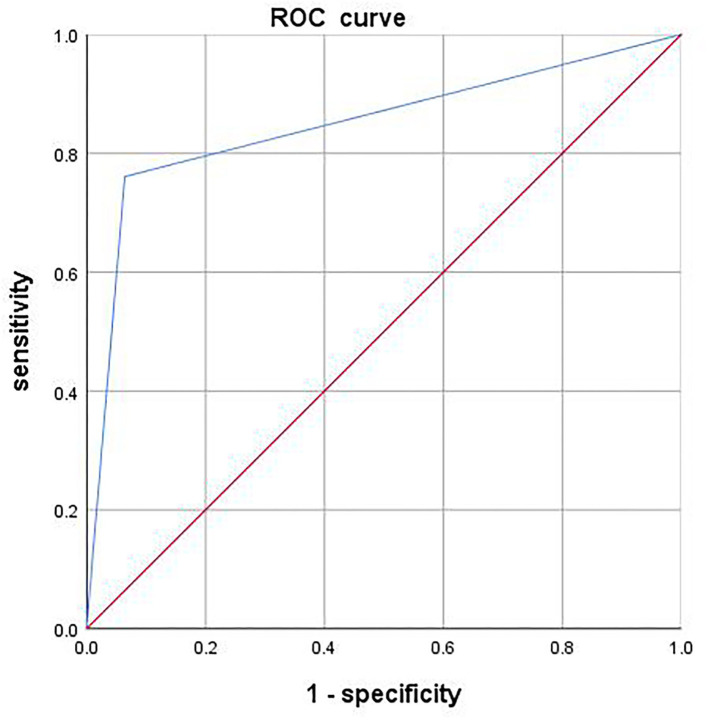
Clinical diagnostic efficacy of the rating questionnaire for screening and early warning of cervical cancer.

See Figure 1 and Table 2.

**Table 2:** Comparison of the coincidence rate, specificity and sensitivity of early warning score diagnosis.

**AUC value**	**S.E**.	**Specificity**	**Sensitivity**	**95% *CI* value**
0.848	0.028	97.22%	86.46%	0.794-0.902

### Comparison of Various High-Risk HPV Infections in Different Cervical Cancer Lesions

See Table 3.

**Table 3:** Comparison of various high-risk HPV infections in different cervical cancer lesions.

**HPV type**	**CINI**	**CINII**	**CINIII**	**Cervical cancer**
16	24	34	26	8
18	27	32	26	7
31	31	25	30	6
33	34	27	26	5
35	36	23	26	7
39	40	30	18	4
45	38	28	19	7
51	35	29	22	6
52	34	32	22	4
56	32	29	26	5
58	36	30	21	5
59	33	28	27	4
66	32	36	18	6
68	29	35	23	5

### Control Effect of the Rating Questionnaire for Screening and Early Warning of Cervical Cancer in Cervical Cancer High-Risk Group

After scientific intervention, HPV test results showed a significant decrease in both high-risk positive cases and low-risk positive cases (*p* < 0.05), and TCT results showed that there was a significant difference in the number of patients with CIN I before and after intervention (*p* < 0.05), and no significant differences in other types were observed (*p* > 0.05). See Table 4.

**Table 4:** Control effect on high-risk population above the threshold [n(%)].

**Time**	**HPV**		**TCT**			
	**High-risk positive**	**Low-risk positive**	**CIN I**	**CINII**	**CINIII**	**Cervical cancer**
Before intervention	35 (38.04%)	57 (61.96%)	28 (30.43%)	31 (33.70%)	24 (26.09%)	9 (9.78%)
After intervention	21 (22.83%)	36 (39.13%)	16 (17.39%)	22 (23.91%)	22 (23.91%)	8 (8.70%)
X^2^	5.031	9.588	4.301	2.417	0.116	0.065
P	<0.05	<0.05	<0.05	0.143	0.733	0.799

## Discussion

Cervical cancer can seriously affect the quality of life of patients, lead to female infertility, and cause damage to the urinary system and vaginal bleeding, and in the late stage of the disease, cancer metastasis may occur, resulting in hydronephrosis as well as uremi. Data show (10, 11) that there are about 110,000 new cervical cancer cases in China each year. Screening of cervical cancer is able to detect precancerous lesions, and when the disease screening coverage rate is over 70%, the incidence and mortality risk of cervical cancer in the population can be effectively reduced. The pathogenesis of cervical cancer is HPV infection, and multiparity and recurrent cervicitis is also the important causes of the high incidence of cervical cancer. The occurrence and development of cervical cancer is a gradual process, from years to decades, which has a long period of reversible precancerous lesions (12). Screening is an effective means to effectively reduce the incidence and mortality of cervical cancer, which can rapidly prevent invasive cervical cancer and precancerous changes in women, and therefore the World Health Organization (WHO) recommends that cervical cancer screening should be initiated worldwide for early diagnosis and early treatment (13). Developed countries have more mature technology and methods of cervical cancer screening and more complete screening programs, so their cervical cancer prevention and control is promising (14, 15). In 2014, HPV DNA typing assay was introduced to China as an initial screening method for cervical cancer, but HPV test cannot determine the specific disease type and single diagnosis has low sensitivity and poor specificity, also, TCT alone has a high rate of missed diagnoses. In addition, with China's large population base and limited health resources, the key point of cervical cancer screening is to develop specific screening programs and select appropriate screening methods (16, 17).

In this study, by reviewing a large number of published works on the epidemiology of cervical cancer and related influencing factors, the Rating Questionnaire for Screening and Early Warning of Cervical Cancer was developed and filled in by women undergoing physical examination, aiming to explore the utility of the questionnaire in diagnosing cervical cancer. Based on the imaging and scoring questionnaires of the study subjects, the specific threshold, i.e., the lowest or highest value an effect can produce, was developed, which helped physicians make a more precise judgment of the occurrence and progression of the disease. The content of the questionnaire included subjects' clinical data, high-risk factors of cervical cancer, and awareness. Previous studies have confirmed (18) that the risk of developing cervical cancer is 2.85 times higher in women having their first sexual intercourse at an age less than 20 years than those having their first sexual intercourse at an age over 20 years, so it is believed that having the first sexual intercourse at younger age is the main reason for the higher incidence of cervical cancer, as well as one of the important factors contributing to the occurrence of the disease in young women. In addition, the risk of cervical cancer in women with more than 3 times of childbirth is 5.91 times higher than that of those with less than 3 times of childbirth, demonstrating that fewer times of childbirth could lower the risk of cervical cancer to some extent (19). And studies also confirmed that low educational degree, poor economic level, poor sanitary conditions in rural areas, etc. are also the high-risk factors triggering cervical cancer. Women with lower education levels lack self-care awareness and gynecological knowledge, and because of their conservative traditional notion, they tend to conceal their illness and avoid treatment, thus increasing the risk of cervical cancer (20). The poor economic level and poor sanitation in rural areas lead to the lack of awareness of regular physical examination among women in these areas, which results in a high incidence of cervical cancer. Therefore, corresponding measures are required to pay close attention to people at high risk of cervical cancer, such as strengthening the publicity of female reproductive health knowledge, conducting targeted implementation of the popularization and publicity of health care knowledge to women, helping women in rural areas get good living habits, strengthening the basic medical and health conditions in township hospitals, putting more efforts in cervical cancer screening for women in rural areas, and assisting women in establishing a correct medical concept (21).

In this study, the diagnostic efficacy of the Rating Questionnaire for Screening and Early Warning of Cervical Cancer in cervical cancer screening was analyzed by plotting ROC curves and taking the results of HPV test and TCT test as the “gold standard”, and the results showed that the AUC value of the questionnaire was 0.848, which indicated a high-clinical application value. In addition, an early warning threshold for the questionnaire was derived in this study by analyzing the questionnaire scores and the gynecological examination findings of the females undergoing physical examination, thereby developing detailed hierarchical interventions based on the threshold. The study implemented scientific intervention measures to those above the threshold, and analyzed and compared the experimental results before and after intervention to explore the control effect. HPV typing assay is designed to target the DNA (the genetic material), through a process of PCR target preference, target amplification, automated real-time detection, and selective amplification, and then identify the corresponding HPV replication with greater precision than second-generation hybrid capture assay. Currently, TCT is the more advanced and mature screening technique for cervical cancer, but it has certain false-negative rate and false-positive rate for low-grade cervical lesions, and the concordance rate increases with higher cytological grade. Scientific means of clinical screening can screen out people at high risk of cervical cancer, reduce the follow-up interval, facilitate effective monitoring of cervical cytological alterations and early detection of cervical lesions, and TCT combined with HPV screening is beneficial to the triage management of cervical lesions, realizing early detection and treatment of the disease. In this study, more detailed prevention and treatment strategies were developed by typing the obtained examination findings. The findings showed that after intervention, both high-risk positive cases and low-risk positive cases were significantly reduced according to the HPV test results (*p* < 0.05), and TCT results denoted that the number of patients with CINI was greatly reduced, which illustrated that the implementation of scientific control measures could lower the risk of cervical cancer to a certain extent. Studies have confirmed (22) that different degrees of TCT and different types of HPV also have significant differences in disease reversal cycles. Data from a large sample survey showed that patients with a TCT result of CIN I and low-risk positive HPV infection had a readily reversible condition, whereas those with a TCT result of CIN II and CIN III and high-risk positive HPV infection required further colposcopy examination and thus had a longer cycle of clinical intervention. Some scholars believe (23) that among multiple high-risk types of HPV, types 16 and 18 have a higher carcinogenic risk, which may be related to the strong ability of gene binding to the host cell, and their gene expression products can also affect the host cell cycle and apoptosis, leading to carcinogenesis. However, other types of viruses are prone to cause low-grade intraepithelial lesions, which may be related to the poor tolerance of the virus to the acidic environment of the vagina, poor viability, and weak pathogenic ability. Type 52 virus has a long cycle of negative conversion and obtains less effective result in clinical treatment; it is also the main genotype responsible for the development of cervicitis. The E6 and E7, biomarkers of cervical cancer caused by type 52 virus, often lead to base mutations that may exert an important function in the progression of cervical intraepithelial lesion and invasive carcinoma, and they are considered as the important carcinogens because they can inactivate the host tumor suppressor p53 and Rb (24).

In addition, unlike the general prevention and control of cervical cancer in developed countries, the comprehensive task of controlling cervical cancer in China is tough and difficult in implementation, and requires long time because of the large population and the uneven development of medical and health resources. Therefore, based on the current situation, the prevention and control measures in China can be summarized as the following 3 aspects. (1) Strengthen the infrastructure construction of the health system, invest corresponding financial, material, and human resources, strengthen the training efforts of primary care workers, and establish a complete system for AI-assisted diagnosis; (2) based on the domestic conditions, clarify the age and screening interval of subjects, identify high-risk individuals, encourage those of proper ages to actively participate in screening, establish a scientific surveillance and evaluation system, and encourage the population of the right age to get an HPV vaccination to gradually expand vaccination coverage; and (3) scientifically develop appropriate screening programs and strategies for the Chinese people to reduce the incidence of cervical cancer (25). Despite the progress of modern medical diagnosis technology, there are still many dilemmas in cervical cancer screening. As medical workers, we still need to continuously try our best to explore more efficient clinical technology, optimize the current diagnosis mode, and provide reliable data support for early screening of cervical cancer. In addition, the application of the Rating Questionnaire for Screening and Early Warning of Cervical Cancer greatly conserves medical and health resources, which is more common and low-cost. The scoring content of the questionnaire should be continually optimized in the future, so that it can be universal in various regions of China as a primary screening method for cervical cancer.

## Data Availability Statement

The original contributions presented in the study are included in the article/supplementary material, further inquiries can be directed to the corresponding author/s.

## Ethics Statement

The studies involving human participants were reviewed and approved by the Ethics Committee of General Hospital of Northern Theater. PLA. The patients/participants provided their written informed consent to participate in this study.

## Author Contributions

DH, BY, and MS: conception and design and data analysis and interpretation. All authors: administrative support, provision of study materials or patients, collection and assembly of data, manuscript writing, and final approval of manuscript.

## Funding

This research was funded by Feasibility study on cervical cancer screening early warning scoring system for female physical examination population in Shenyang, Grant No. 20-205-4-088.

## Conflict of Interest

The authors declare that the research was conducted in the absence of any commercial or financial relationships that could be construed as a potential conflict of interest.

## Publisher's Note

All claims expressed in this article are solely those of the authors and do not necessarily represent those of their affiliated organizations, or those of the publisher, the editors and the reviewers. Any product that may be evaluated in this article, or claim that may be made by its manufacturer, is not guaranteed or endorsed by the publisher.
